# ARIZONA study: is the risk of post-herpetic neuralgia and its burden increased in the most elderly patients?

**DOI:** 10.1186/1471-2334-14-529

**Published:** 2014-10-01

**Authors:** Martin Duracinsky, Marc Paccalin, Gaëtan Gavazzi, Sohéla El Kebir, Jacques Gaillat, Christophe Strady, Didier Bouhassira, Olivier Chassany

**Affiliations:** Paris-Diderot University Sorbonne Paris Cité, EA 7334 REMES, Patient-Reported Outcomes Unit, 75010 Paris, France; Internal Medicine and Clinical Immunology Department, AP-HP, Bicêtre Hospital, 94275 Cedex Le Kremlin-Bicêtre, France; Department of Geriatrics, La Milétrie Hospital, University Hospital of Poitiers, 86000 Poitiers, France; Geriatric Medicine Department, A. Michallon North Hospital, University Hospital, 38700 La Tronche, France; Mapi, Real World Evidence, 69003 Lyon, France; Infectious Diseases Department, Annecy Regional Hospital, 74374 Pringy, France; Cabinet d’infectiologie, Clinique Saint André – Groupe Courlancy, 5 Boulevard de la paix, 51100 Reims, France; INSERM U987, Pain Evaluation and Treatment Centre, Ambroise Paré Hospital, 92100 Boulogne-Billancourt, France; Versailles Saint-Quentin en Yvelines University, 78000 Versailles, France; Clinical Research and Development Department, AP-HP, Saint-Louis Hospital, 75010 Paris, France

**Keywords:** Elderly, Herpes zoster, Post-herpetic neuralgia, Neuropathic pain, Quality of life

## Abstract

**Background:**

In a context of change in the demographic profile of the older population, to identify an age threshold for increased risk and burden of herpes zoster (HZ) in 70+ patients.

**Methods:**

*Post hoc* analysis of the 12-month French nationwide prospective observational ARIZONA cohort study. HZ was assessed by means of the following validated questionnaires: Neuropathic Pain Symptom Inventory (NPSI), Zoster Brief Pain Inventory (ZBPI), Short-Form health survey (SF-12), and Hospital Anxiety and Depression Scale (HADS).

**Results:**

644 general practitioners included 1,358 volunteer patients with acute HZ in the ARIZONA study; 609 patients (45%) were 70+. In 70+ patients, age did not increase rash severity or HZ-related pain intensity at diagnosis, but increased by 64% the frequency of ophthalmic zoster (from 5.5% in 70–74 years age-group to 9.0% in 85+ patients, p = NS). Age was significantly associated with low physical health as assessed by the SF-12 Physical Component Summary (SF-12 PCS) score and bad mood as assessed by the HADS depression score (p < 0.001). Within the year following HZ, post-herpetic neuralgia (PHN) was systematically but not significantly more frequent in 85+ patients than in the 70–74, 75–79, or 80–84 years age-groups (19.0% *vs*. 13.3%/15.3%/11.6% at month 3; 15.1% *vs.* 7.3%/11.0%/12.2% at month 6; 15.2% *vs.* 6.0%/8.0%/6.0% at month12, respectively). SF-12 PCS and HADS depression scores improved from day 0 to month 12 in all patients (p < 0.001). 85+ patients were more impaired than younger patients (p < 0.001), but without clear difference according to PHN.

**Conclusions:**

This study did not show in 70+ patients a clear and significant age threshold at which disease burden increased, although for some domains the impact seemed higher among the oldest patients; the cut-off of 70 years remains thus relevant for clinical and epidemiological studies. However, at individual level, assessment of the burden of HZ and HZ-related pain appears necessary to improve management and prevent functional decline in the most vulnerable 70+ patients.

**Electronic supplementary material:**

The online version of this article (doi:10.1186/1471-2334-14-529) contains supplementary material, which is available to authorized users.

## Background

Herpes zoster (HZ) results from reactivation of varicella-zoster virus (VZV) from a latent infection in the sensory ganglia [1, 2]. HZ affects peripheral nerves and induces painful skin and nerve lesions. The acute phase is usually defined as ≤1 month after rash onset, while post-herpetic neuralgia (PHN), the most common complication, is often defined as pain persisting for ≥3 months after rash onset [3].

In part due to the decline of cell-mediated immunity with age, age is the major risk for developing both HZ and HZ-related pain, including PHN [4–7]. Previous studies suggested that HZ and PHN substantially impair health-related quality of life (HRQoL), that PHN may reduce the ability to maintain an independent lifestyle, and that acute and chronic HZ-related pain may impair activities of daily living, psychological well-being and social interactions [8–13]. The real long-term burden of illness experienced by HZ patients, and in particular by older patients, is not clearly known due to a lack of large-scale longitudinal studies [14]. Such data are needed with the expected increase in the number of people who will develop HZ and HZ-related pain [11–15].

ARIZONA is a large prospective longitudinal cohort study aiming to assess the real-life burden of HZ from the patient’s perspective and the influence of age on disease burden. It was conducted in France in patients aged 50-years or more (50+) with acute HZ in the eruptive phase. Its main results have been recently published [16]. Multivariate analysis of ARIZONA results has shown that age 70+ was an independent predictor of PHN at 3 months and that HZ-related pain, including PHN, impaired daily life functioning, HRQoL and mood. The age cut-off (70 years) was selected for consistency with the efficacy analysis of the Shingles Prevention Study, which compared the incidence of HZ and PHN in two groups receiving shingles (HZ) vaccine or placebo [17].

The world population is ageing. Moreover, the demographic profile of the older population is changing: the proportion of persons aged 80 or over (80+) is projected to increase almost fourfold over the next 50 years [18]. In this context, to determine an age threshold at which HZ and PHN are particularly at risk for patients, including beyond 70 years of age could be helpful to improve patients’ management.

The objective of this complementary analysis, performed on data from the most elderly ARIZONA patients (i.e., 70+ only), was thus to determine whether, beyond 70 years, age remained a risk factor for PHN and increased the burden of the disease, and whether a new age threshold could be defined in the most elderly patients.

## Methods

The longitudinal prospective multicentre observational ARIZONA study was conducted in general practices in France between 20 November 2006 and 12 September 2008. The study, managed by a multidisciplinary scientific committee, was carried out in accordance with the principles of the Declaration of Helsinki (2004); approval was provided by an independent French review board (CCTIRS for *Comité consultatif sur le traitement de l’information en matière de recherche*). All patients provided written informed consent before enrolment. The study methods were published in detail in 2012 [16].

Briefly, 644 of the 29,177 general practitioners (GPs) randomly selected from the list of GPs practicing in France and contacted by post agreed to participate in the study and included at least one patient. Each GP included consecutively all 50+ patients with acute HZ in the eruptive phase if (1) they were seen within 7 days of rash onset and (2) they had good understanding of French and telephone access. Eruptive phase was defined as visible skin lesions at any stage of development. Patients with HZ in the preceding 12 months or participating in any clinical trial were excluded. The study was managed by a multidisciplinary scientific committee. The protocol was approved by an Ethics Committee. All patients gave written informed consent.

At the inclusion consultation (day 0), the GP documented the subject’s demographic and medical characteristics, HZ characteristics, and currently prescribed drugs. Rash severity was assessed by the GP based on quantitative and qualitative assessment of lesions.

The baseline questionnaire was to be completed by the patients at the GP’s office during the recruitment consultation or at home within a day of the consultation. It comprised a dedicated form assessing the patient’s perception of HZ-related pain and four validated self-reported questionnaires: the Neuropathic Pain Symptom Inventory (NPSI) [19]; the Zoster Brief Pain Inventory (ZBPI) [9, 20]; the 12-item Short-Form health survey (SF-12) [21]; and the Hospital Anxiety and Depression Scale (HADS) [22].

The NPSI assesses the severity of each of the 10 symptoms of neuropathic pain on a scale ranging from 0 (no pain) to 10 (worst pain imaginable) [19]. On the global NPSI score, mild pain is defined by scores ranging between 0 and 29, moderate pain by scores ranging between 30 and 79, and severe pain by scores ranging between 80 and 100. The ZBPI rates the severity of pain on a scale ranging from 0 (no pain) to 10 (pain as bad as you can imagine), and the interference of pain on the activities of daily life on a scale ranging from 0 (does not interfere) to 10 (completely interferes); mild interference is defined by scores ranging between 0 and 2, moderate interference by scores ranging between 3 and 4, and severe interference by scores ranging between 5 and 10 [9, 20]. The Physical Component Summary (PCS) score and the Mental Component Summary (MCS) scores were calculated from the SF-12 [21]. SF-12 PCS or MCS scores <50 indicates HRQoL impairment (range score: 0–100). HADS anxiety or depression scores above 8 indicate anxiety or depression [22].

During the follow-up period (months 3, 6 and 12), patients were contacted by telephone and completed a follow-up questionnaire with the help of a trained interviewer, comprising the same dedicated form and questionnaires. PHN was defined as the persistence of pain of any intensity 3 months or more after the rash onset.

All statistical analyses were performed using version 8.02 SAS software (SAS Institute, Inc., Cary, NC, USA). For this complementary analysis, quantitative variables were described by frequency, mean, standard deviation (SD), median and range, according to four (70–74, 75–79, 80–84, and 85+ years) and two (70–79 and 80+ years) age-groups, or to two groups defined by PHN (with or without) at months 3, 6, and 12. The four age-groups have been defined based on the 85+ age-group (i.e., currently considered as the ‘old old’) in order to have a similar number of patients in each age-group. Results based on the two age-groups are disclosed only when they brought additional information to the comparison made on the four age-groups. Qualitative variables were described by frequency and percentage of each modality. Fisher, Chi^2^, or Wilcoxon tests were used to compare groups. Regression analysis for repeated measures was used to assess the impact of age on score evolution over time (day 0 and months 3, 6 and 12) and the influence of age on each parameter regardless of time.

## Results

### Participants

644 GPs enrolled in the ARIZONA study 1,517 patients aged 50 years and over, of whom 1,358 satisfied the inclusion criteria. Out of these 1,517 patients, 609 (45%) were 70+ and were included in the present complementary analysis: 383 patients (62.9%) were between 70 and 79 years of age and 226 (37.1%) were 80+; respectively, 220 (36.1%), 163 (26.8%), 137 (22.5%), and 89 patients (14.6%) belonged to the 70–74, 75–79, 80–84, and 85+ age-groups.

Table 1 presents the main baseline characteristics of the elderly patients in each of the four age-groups. In all age-groups, there were more women than men. Comorbidity (mainly cardiovascular disease) was frequent in all age groups (>70%).

**Table 1:** **Main baseline characteristics of patients (70+) included in the ARIZONA study**

Age (years)	70-74 (N = 220)	75-79 (N = 163)	80-84 (N = 137)	85+ (N = 89)	***p- value***
**Sex (female): %**	60.9	61.3	61.0	76.1	*0.062*
**BMI (kg/m** ^**2**^ **): mean (SD)**	26.6 (4.8)	26.6 (4.6)	24.9 (4.0)	25.4 (4.4)	*0.001*
**Comorbidity: %**	72.8	78.4	81.5	78.7	*0.261*
Cardiovascular disease	63.3	73.2	80.9	78.6	*0.008*
Diabetes	13.3	17.3	4.5	11.4	*0.324*
Cancer	8.2	11.0	8.6	11.0	*0.263*
Chronic pulmonary disease	10.1	12.6	10.9	14.3	*0.801*
Other chronic disease	33.5	24.4	28.2	25.7	*0.351*
**Previous history of VZV disease: %**					
Known history of varicella	51.1	48.1	41.6	45.5	*0.642*
Known history of Herpes zoster	20.5	17.9	16.1	11.2	
***If history of VZV disease: %***					*0.663*
≥12 and <24 months	9.3	10.3	4.5	0.0	
>24 months	90.7	89.7	92.5	100.0	
**Familial status: %**					*<0.001*
Married or partner	67.9	63.4	45.5	32.4	
Widowed	21.2	26.1	43.0	59.5	
Divorced or separated	6.2	7.0	5.0	2.7	
Single	4.7	3.5	6.6	5.4	
**Living conditions: %**					*<0.001*
At home or with close relatives	96.9	97.9	91.7	82.7	
In a community	3.1	2.1	8.3	17.3	

*BMI* = body mass index; *SD* = standard deviation; *VZV* = varicella zoster virus.

### HZ characteristics at baseline

Table 2 presents HZ characteristics at baseline. The time-interval between rash onset and HZ diagnosis was commonly ≤2 days in all age-groups. Rash severity did not qualitatively or quantitatively increase with age after 70 years. Ophthalmic zoster location was more frequent in the most elderly patients although the difference between age-groups was not significant (p = 0.57): the frequency of ophthalmic zoster ranged from 5.5% in the 70–74 age-group to 9.0% in the 85+ age-group.

**Table 2:** **Main baseline characteristics of herpes zoster and herpes zoster-related pain in each age-group**

Age group (years)	70-74 (N = 220)	75-79 (N = 163)	80-84 (N = 137)	85+ (N = 89)	***p-value***
**Time-interval between rash onset and herpes zoster diagnosis: %**					*0.179*
≤1 day	46.3	34.6	41.9	37.9	
>1 and ≤2 days	22.7	22.2	24.3	31.0	
>2 and ≤3 days	14.8	16.0	14.0	16.1	
>3 days	16.2	27.2	19.9	14.9	
**Rash location: %**					*0.070*
Cranial, cervical, facial, and/or ophthalmic	15.0	16.6	19.0	28.1	
Thoracic, abdominal and/or sacrolumbar	72.3	66.3	62.8	61.8	
Upper and/or lower limbs	8.2	11.7	8.0	5.6	
Other location and/or combination of previous locations	4.5	5.5	10.2	4.5	
**Rash severity (quantitative assessment): %**					*0.775*
Few vesicles	43.9	38.9	43.5	41.2	
Many vesicles (extensive rash)	56.1	61.1	56.5	58.8	
**Rash severity (qualitative assessment): %**					
Simple vesicles	86.9	80.3	85.5	81.2	*0.294*
Haemorrhagic lesions	7.9	6.4	7.6	12.9	*0.328*
Necrotic lesions	3.7	12.1	6.1	4.7	*0.650*
Ophthalmic zoster	5.5	8.0	8.8	9.0	*0.574*
**Time interval between pain and HZ vesicles onset: mean (SD) (days)**	−1.0 (2.3)	−0.9 (2.5)	−0.8 (2.4)	−0.7 (2.1)	*0.639*
NPSI*: mean (SD)	32.7 (18.5)	29.9 (21.6)	31.1 (18.5)	33.3 (18.0)	*0.407*
**ZBPI**†**: worst pain severity score, mean (SD)**	5.4 (2.5)	5.1 (2.8)	5.8 (2.6)	5.5 (2.6)	*0.185*
**Medical treatment: yes (%)**	97.7	98.2	100	98.9	*0.337*
Antiviral drug	96.3	93.1	92.7	96.6	*0.318*
Analgesics	85.6	86.3	88.3	86.4	*0.907*

*NPSI* = Neuropathic Pain Symptom Inventory; *SD* = Standard deviation; *ZBPI* = Zoster Brief Pain Inventory.

*NPSI score > 30 indicates moderate pain.

^**†**^from 0, no pain to 10, maximal pain.

HZ-related pain, whose onset always slightly preceded rash onset in all age-groups, was frequent at baseline (reported by about 80% of patients, n = 486). It was of moderate intensity without difference between age-groups: mean NPSI score around 30 and mean worst pain ZBPI severity score around 5 in all age-groups (Table 2). Burning pain was the main descriptor (data not shown).

98.5% of patients had at least one medical prescription, mainly antiviral drugs (94.7%) and analgesics (86.5%). The percentage of patients under treatment did not differ with age.

### Impact of HZ and HZ-related pain on daily life, HRQoL, and mood at baseline

Results are presented in Table 3. At baseline, HRQoL, and mood were impaired in all age-groups: mean ZBPI interference scores were around 3, mean SF-12 PCS and MCS scores were below 50, and mean HADS depression scores close to 8.

**Table 3:** **Burden of herpes zoster and herpes zoster-related pain at baseline in each age-group**

Age group (years)	70-74 (N = 220)	75-79 (N = 163)	80-84 (N = 137)	≥85 (N = 89)	***p-value***
**ZBPI score: mean (SD)**					
Interference score*	3.2 (2.3)	3.1 (2.4)	3.5 (2.3)	3.6 (2.7)	*0.582*
**SF-12**†					
PCS score: mean (SD)	42.7 (8.9)	41.8 (9.4)	37.9 (10.0)	34.2 (8.9)	*<0.001*
Physical functioning	57.9 (35.3)	53.8 (34.9)	40.7 (34.2)	26.6 (33.6)	*<0.001*
Vitality	36.6 (25.7)	33.5 (23.0)	28.8 (23.5)	25.4 (23.0)	*0.004*
MCS score: mean (SD)	41.1 (10.2)	38.7 (11.5)	39.6 (10.9)	41.0 (9.8)	*0.413*
**HADS: mean (SD)** ^**‡**^					
Depression score	6.3 (4.0)	7.9 (4.4)	8.1 (5.2)	9.8 (4.9)	*0.001*
Anxiety score	6.9 (4.2)	7.9 (4.1)	7.3 (4.6)	7.1 (4.3)	*0.237*

*HADS* = Hospital Anxiety and Depression Scale; *MCS* = Mental component summary; *PCS* = Physical component summary; *SD* = Standard deviation; *SF-12* = 12-item Short-Form health survey; *ZBPI* = Zoster Brief Pain Inventory.

*From 0, no interference to 10, maximal interference.

^**†**^Scores < 50 indicates health-related quality of life (HRQoL)impairment.

^**‡**^Scores > 8 indicate probable anxiety or depression.

No significant difference was found in mean ZBPI interference scores between the four age-groups (p = 0.582).

As regards HRQoL, SF-12 PCS scores decreased significantly with age (p < 0.001), indicating greater HRQoL impairment in the most elderly patients. The low SF-12 PCS scores observed in the 85+ age-group were mainly due to the scores of the ‘physical functioning’ and ‘vitality’ dimensions. No statistically significant difference in SF-12 MCS scores was observed between the four age groups (p = 0.413).

As regards mood, HADS depression scores increased significantly with age (p < 0.001), indicating greater mood impairment in the most elderly patients. No statistically significant differences in HADS anxiety scores were observed between the four age groups (p = 0.237).

### Change in HZ-related pain, daily life, HRQoL, and mood from day 0 to month 12

**Figure 1:**
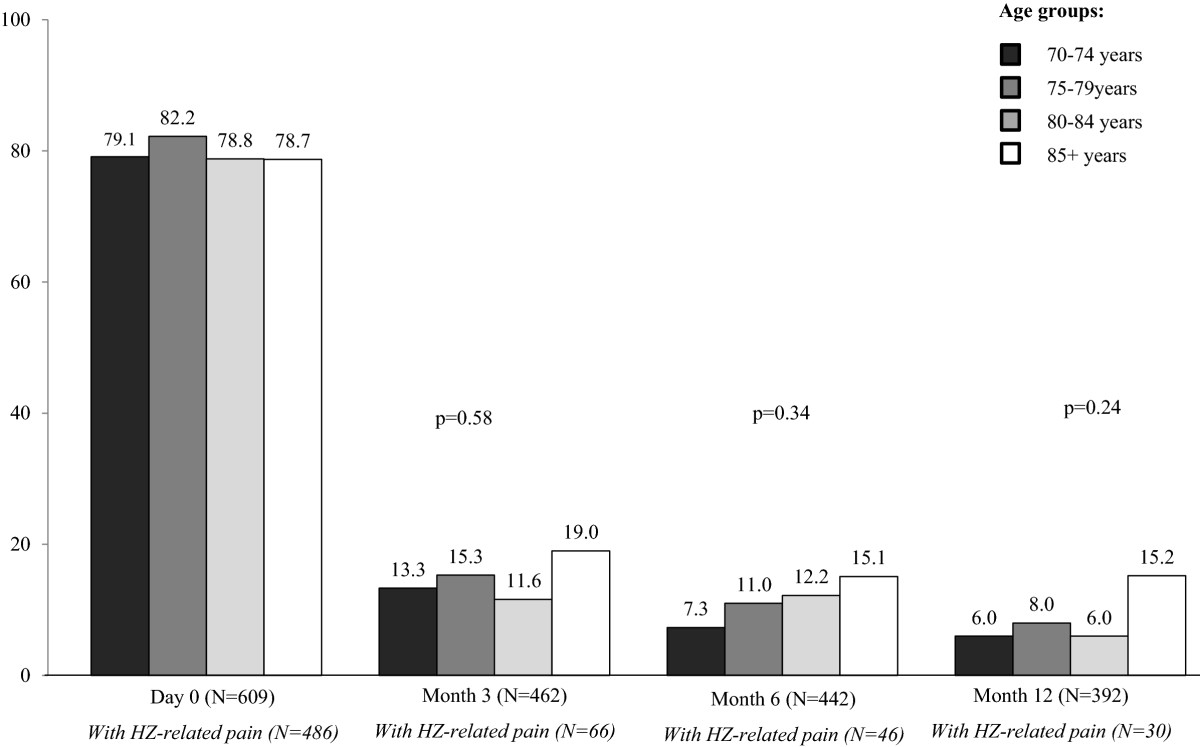
**Percentage of patients with herpes zoster (HZ)-related pain during the 12-month follow-up period in each age-group.** Percentages were calculated on available data.

**Figure 2:**
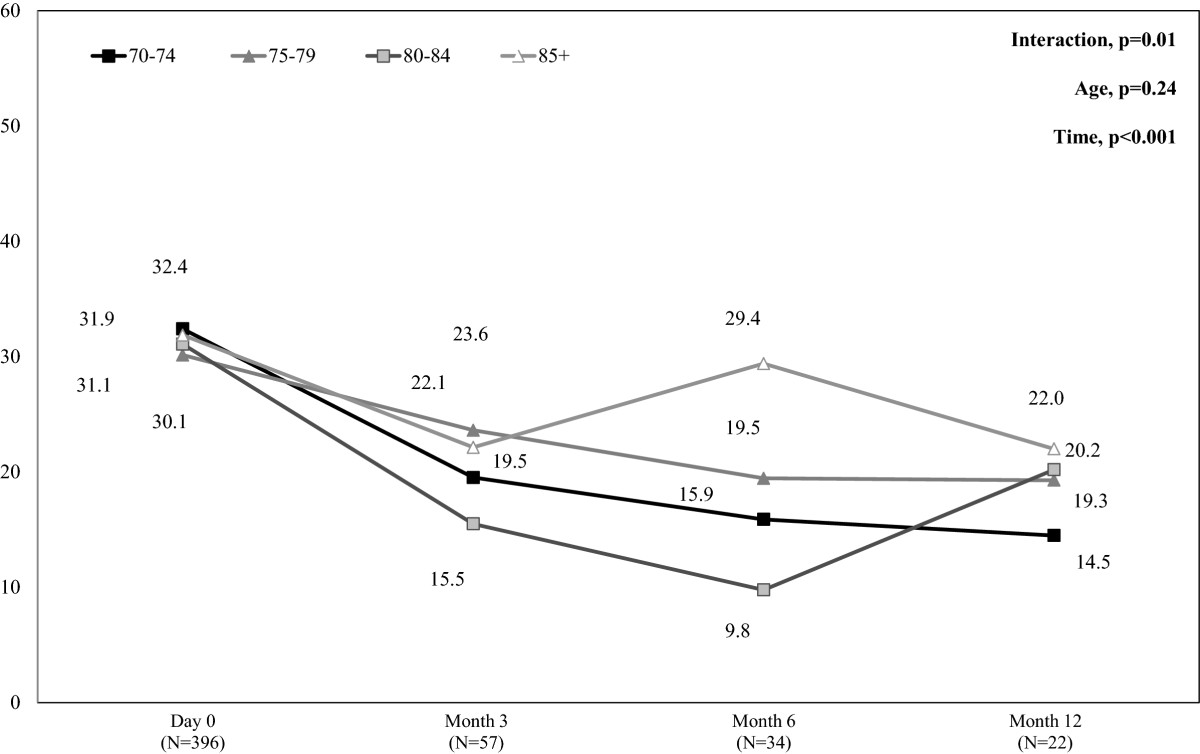
**Mean NPSI scores in patients with herpes zoster (HZ)-related pain during the 12-month follow-up period by age-group.** NPSI = Neuropathic Pain Symptom Inventory. NPSI score >30 indicates moderate pain (range score 0–100). Mean NPSI scores were calculated in the population of patients aged 70 years or more with HZ-related pain (day 0) or PHN (months 3, 6, and 12) and available NPSI data at each time-point. Missing data were not replaced.

Figure 1 presents the percentage of patients who reported HZ-related pain from day 0 to month 12. Prevalence of HZ-related pain dramatically decreased between day 0 and month 3 in all age-groups: overall, 66 patients (14.3%) had PHN at month 3. Prevalence of PHN slightly decreased from months 3 to 12: 30 patients (7.6%) had PHN at month 12. At months 3, 6 and 12, the percentage of patients with PHN was always higher in the 85+ than in the other age-groups. However, no statistically significant difference was found between the four age-groups (p = 0.58, p = 0.34, and p = 0.24, respectively).Figure 2 presents mean NPSI scores from day 0 to month 12 in each age-group in patients who reported HZ-related pain (day 0) or PHN (months 3, 6 and 12). Pain intensity assessed by NPSI decreased significantly between day 0 and month 3, and then decreased slightly between months 3 and 12 in all age-groups except for the 85+ age-group at month 6 and the 80–84 age-group at month 12, leading to a statistically significant interaction between time and age (p = 0.01).

**Figure 3:**
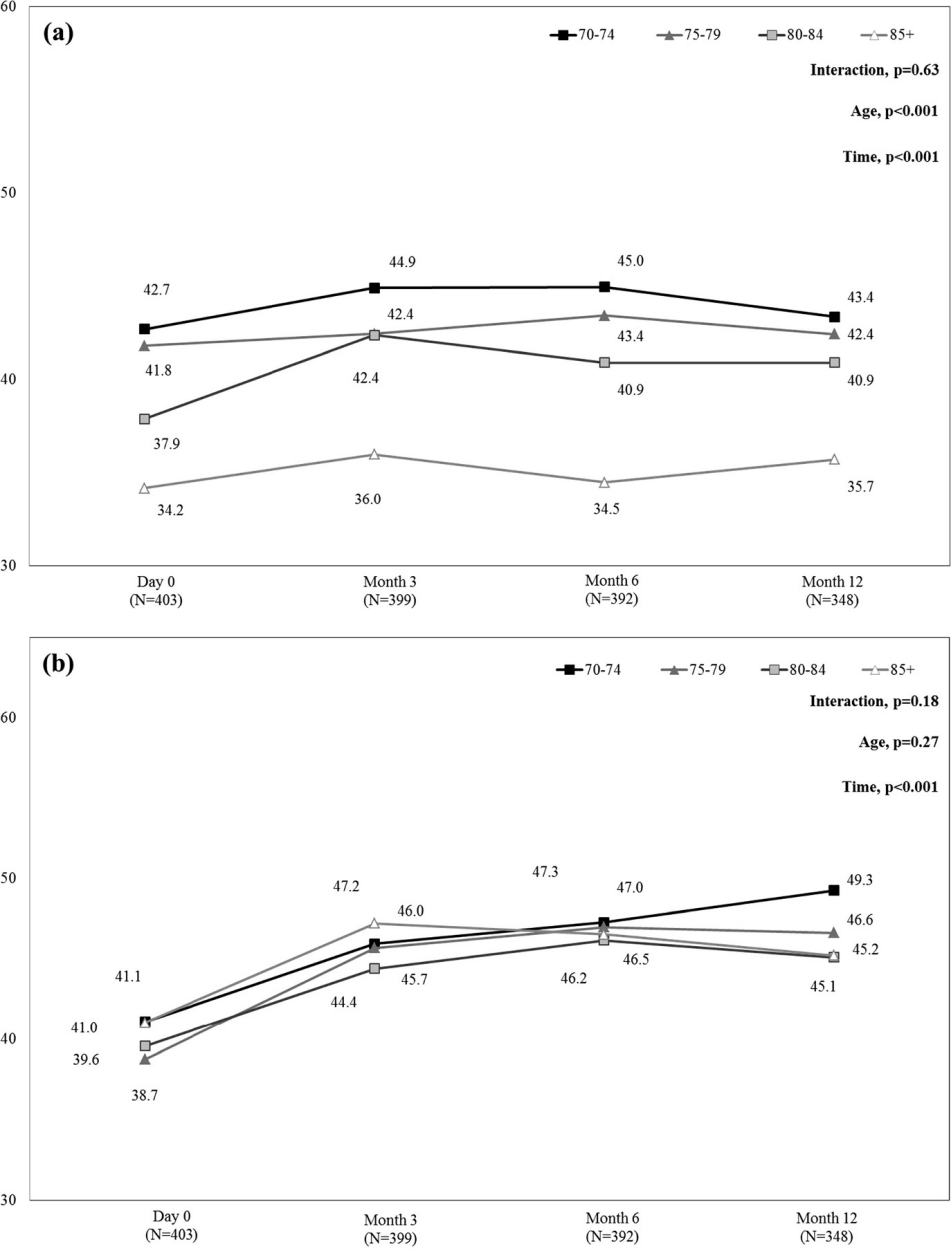
**Mean SF-12 PCS (a) and MCS (b) scores during the 12-month follow-up period by age-group.** MCS = Mental Component Summary; PCS = Physical Component Summary; SF-12 = 12-item Short-Form health survey. SF-12 MCS or PCS score <50 indicates quality of life impairment [mean (standard deviation) score in the general 1998 U.S. population was 50 (10)] [12]. Mean MCS and PCS scores were calculated in the whole population of patients aged 70 years or more with available data at each time-point. Missing data were not replaced.

**Figure 4:**
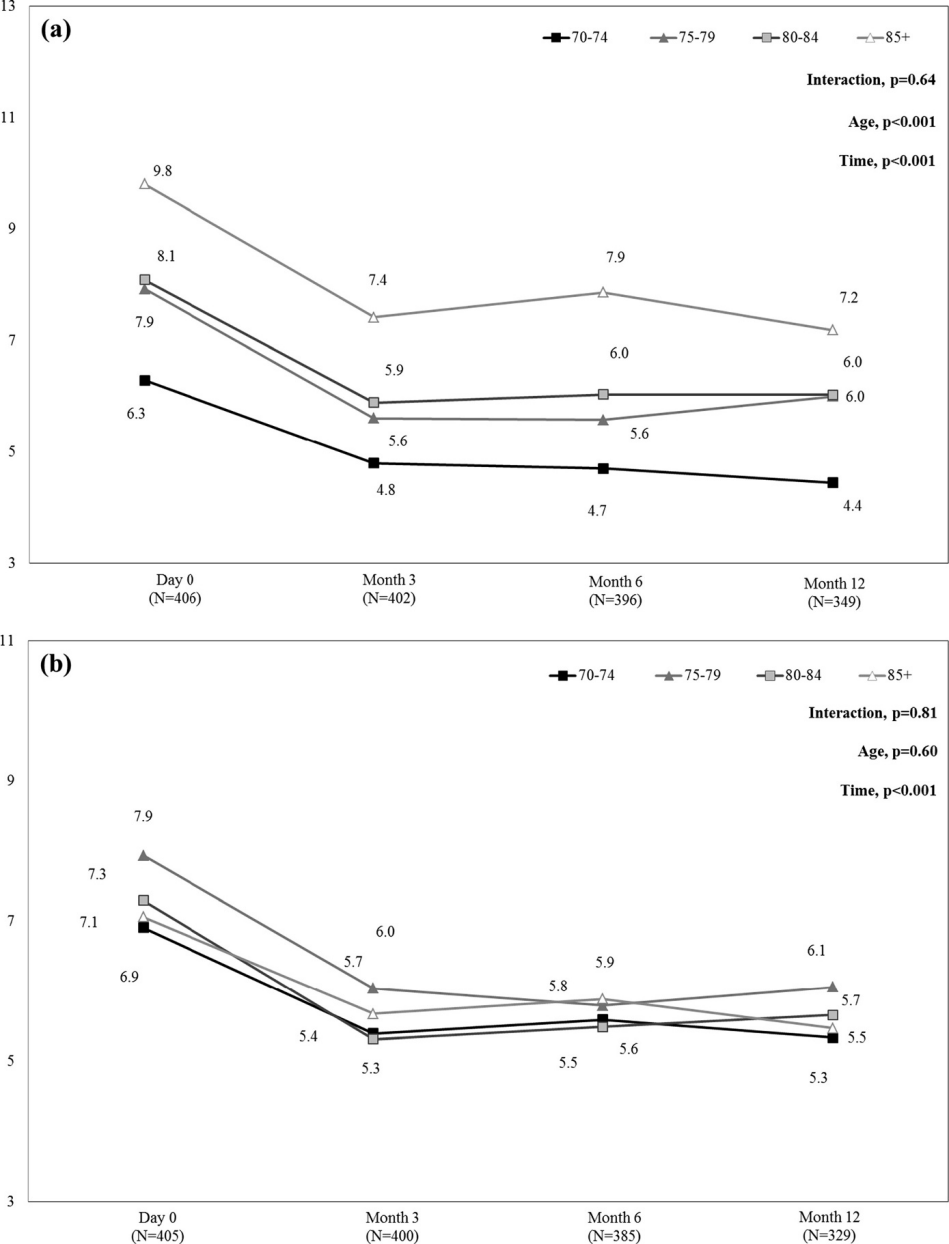
**HADS depression (a) and anxiety (b) scores during the 12-month follow-up period by age-group.** HADS: Hospital Anxiety and Depression Scale. Score >8 indicates depression or anxiety. HADS scores were calculated in the whole population of patients aged 70 years or more with available data at each time-point. Missing data were not replaced.

During follow-up, ZBPI interference scores in patients with HZ-related (day 0) or PHN (months 3 to 12) remained high (around 3), with no difference between age-groups (data not shown).Figure 3 presents mean SF-12 PCS and MCS scores from day 0 to month 12 in each age-group. SF-12 PCS scores increased slightly, from day 0 to month 12, in all age groups (p < 0.001), indicating slight improvement in HRQoL. These results were always driven by the ‘physical functioning’ and ‘vitality’ scores (data not shown). In addition, at each time point, SF-12 PCS scores were lower in the 85+ age-group than in the other age-groups (p < 0.001), indicating greater HRQoL impairment in the most elderly patients. Comparison within the 85+ age-group found a significant difference in SF-12 PCS scores between patients with and without PHN at month 3 (mean +/− SD: 27.0 +/− 11.26 and 37.6 +/− 12.26, respectively; p = 0.015), but not at months 6 and 12 (p = 0.956, and p = 0.919, respectively), indicating that impact of age and its comorbidities prevails over consequences of PHN. Regarding SF-12 MCS summary scores, from day 0 to month 12, SF-12 MCS scores increased in all age-groups (p < 0.001), indicating an improvement in HRQoL, without statistically significant difference in SF-12 MCS scores according to age (p = 0.27).Figure 4 presents mean HADS depression and anxiety scores from day 0 to month 12 in each age-group. HADS depression scores decreased from day 0 to month 12, in all age-groups (p < 0.001), indicating mood improvement. In addition, at each time point, HADS depression scores were greater in the 85+ age-group than in the other age-groups (p < 0.001), indicating greater mood impairment in the most elderly patients. Comparison within the 85+ age-group found no significant difference in HADS depression scores between patients with and without PHN at months 3, 6, and 12 (p = 0.52, p = 0.83, and p = 1.00, respectively), indicating that the impact of age prevails over consequences of PHN. However, when patients were classified into two age-groups, in the 80+ age-group, HADS depression scores significantly differed between patients with and without PHN at months 3 and 12 (p = 0.01, and p = 0.04, respectively) but not at month 6 (p = 0.30). HADS anxiety scores decreased from day 0 to month 12, in all age-groups (p < 0.001), indicating mood improvement, without statistically significant difference according to age (p = 0.60).

## Discussion

In the ARIZONA study, which is to our knowledge the largest and longest prospective study to assess HZ burden in patients aged 50 years or more (50+), it was previously shown that age ≥70 years was a risk factor for both PHN and impaired HRQoL [16]. In a context of ageing of the older European population, the objectives of this *post hoc* analysis of the data collected during the ARIZONA study was to determine whether, beyond 70 years, age remained a risk factor for PHN and increased the burden of the disease, and whether a new age threshold could be defined in the most elderly patients.

### Frequency and severity of pain during the acute phase of HZ infection

Older age has long been considered as a risk factor for severe pain during the acute phase of HZ infection [3]. In the entire ARIZONA cohort, we showed that at baseline the proportions of patients with extensive rash or haemorrhagic or necrotic lesions were higher in patients 70+. By contrast, no difference between groups of patients was observed in the frequency or severity of HZ-related pain at inclusion [16]. In the MASTER study [23–25], older age also ‘emerged as an independent predictor of greater severity-of-illness over 180 days, but not during the first 90 days after rash onset’. According to the authors, this result indicated that younger patients were as likely as older patients to experience considerable burden during the acute phase of HZ, but had a lower risk of developing PHN and consequently experiencing lower severity-of-illness over the entire episode. The present results confirm that, in patients 70+, the incidence of HZ-pain during the acute phase was large and similar in all age groups: regardless of age, approximately 80% of them reported HZ-related pain. Our data also indicate that there is no specific age cut-off after 70 years of age at which the frequency or intensity of pain during the acute phase of HZ infection is further increased. However, this analysis shows that the ophthalmic location of HZ infection tended to increase with age, from 5.5% in the 70+ group to 9% in the 85+ group.

### Frequency and severity of PHN

Depending on the time-frame used in the literature, PHN occurs in 5% to more than 30% of patients [26]. Several possible risk-factors such as age, immunodepression, prodromal pain, and ophthalmic zoster have been considered [25]. PHN, defined as HZ-related pain persisting ≥3 months, was reported by about 30% of 70+ patients in an Icelandic study [27], 13% of 70+ patients in the ARIZONA study, and 45% of patients in the MASTER study [23–25]. After one year, 15% of 70+ patients in the Icelandic and 8% of the same age class in the ARIZONA study still had pain. The percentage of subjects with PHN was lower in the ARIZONA study than in the MASTER study. In the ARIZONA study [16], however, antiviral drugs were prescribed to 94.1% of patients for a median 7 days, which may have contributed to reduce the intensity of acute pain, accelerate vesicular rash healing and reduce acute-phase viral excretion duration [28]; conversely, in the MASTER study [23], only 26% of included patients received antiviral medication at the recommended dose and time. Although the efficacy of antiviral medication in PHN prevention is controversial [29], it may thus be hypothesised that early initiation of adequate treatment may have contributed to the lower prevalence of related pain at 3 months as compared with the MASTER study [23–25]. These findings nevertheless support the need for preventive vaccine strategies in elderly patients since more than 1 in 10 patients will develop PHN even with adequate treatment. Vaccination was shown to reduce the incidence of HZ and PHN and the HZ burden of illness (a composite end point sensitive to the incidence, severity and duration of HZ pain), to have a good safety profile [17, 30], and to be cost effective in elderly patients [31, 32]. Centers of Disease Control and Prevention (CDC) recommended HZ vaccine for prevention of HZ and its complications among adults aged 60 years or more [33].

According to the literature, older age is an independent factor of PHN [3–7]. The ARIZONA study showed that older age (70+) at baseline was an independent predictive factor for PHN [16]. Severity of HZ-related pain was comparable for 70+ patients and younger, but the prevalence was significantly higher for 70+ patients at months 1, 3, 6, and 9 and close to significance at month 12. The present data showed that the percentage of patients with persistent HZ-related pain at months 3, 6, and 12 was systematically but not statistically significantly higher in the most elderly patients (i.e. 85+).

Pain intensity appeared to remain constant in patients with persistent HZ-related pain (regardless of age). This additional result was partly consistent with that of the Canadian MASTER study, which found that mean pain intensity decreased during the first month of the disease and then remained stable from months 1 to 6 [23]. In addition, no clear difference in the severity of PHN was observed according to age. This can possibly be partly explained by a negative influence of age on perceived degree of pain, as shown in acute myocardial ischemia as being a possible cause for delayed treatment in elderly patients, with a similar cut-off at 69 years [34].

### Disease burden

It has been previously described that the PCS score of the SF-12 shows impairment with age, and that HZ infection severely impacts HRQoL [35, 36]. In addition, it has been recently showed that an episode of HZ could lead to comorbidity decompensation and could jeopardize the health status of an older person with concomitant diabetes, COPD, or cardiovascular disease [37]. An impairment of HRQoL due to both age and PHN could thus be expected in the oldest patients in the ARIZONA study. In fact, only the physical component of HRQoL as assessed by the SF-12 PCS and depression as assessed by the HADS were impacted by age. Indeed, the difference over 8 points of baseline SF-12 PCS score between 85+ and 70–74 age groups exceeds the minimal important difference of 3 points, by a meaningful [12].

This study showed that the older the patient, the lower the SF-12 PCS score and the higher the depression HADS score but failed to find clear difference in SF-12 PCS and HADS depression scores between patients with and without PHN at months 3, 6, and 12. It may be thus suggested that the impairment of HRQoL was related more to age and its comorbidities than to the HZ infection *per se*.

However, the absence of significant difference can also be explained by some of the limitations in this our study. A selection bias cannot be ruled out as only data from patients who agreed to participate in the study were collected. However, the mean number of included patients per GP was consistent with the mean number of patients seen in general practice for HZ according to the French GPs Sentinelles network (https://websenti.u707.jussieu.fr/sentiweb/?page=presentation), indicating that almost all patients seen for HZ by a GP agreed to participate in the study. The second limitation is the small sample size and heterogeneity of the different age-groups (although as a whole, the sample size of ARIZONA cohort was large, with over 1,350 patients), which may not be sufficient to detect any effect. However, similar results were usually observed when patients were classified into two larger age-groups (70–79 and 80+). Finally, although the frailty risk increases with age, the population of 70+ patients is probably highly heterogeneous: both well and frail patients with various chronic diseases are included in each age-group. That is why it is difficult to separate influence of HZ and age and chronic diseases. Further longitudinal studies with a large number of very old patients assessing chronological and physiological ages or frailty are thus needed to evaluate HZ and PHN burden in the 70+. Indeed, beyond the age of 70 years, HZ and PHN can impair frail patients. In the model by Rockwood et al., frailty can be thought of as a dynamic balance between assets which help maintain a person’s independence and deficits which threatens a person’s self-sufficiency or functional capacity. In frail elderly, HZ and PHN can tip the balance in favour of the deficit [38].

## Conclusion

There is no definite age threshold at which HZ severity, PHN frequency, pain intensity or disease burden as assessed by validated scales increased in patients aged 70 years or more. At population level, the cut-off of 70 years remains thus relevant for clinical and epidemiological studies. However, beyond the age of 70 years, HZ and PHN can impair frail patients. At individual level, assessment of the burden of HZ, HZ-related pain, and PHN on daily life, quality of life and mood in elderly patients appears necessary to improve their management and prevent functional decline.

## Authors’ original submitted files for images

Below are the links to the authors’ original submitted files for images. Authors’ original file for figure 1 Authors’ original file for figure 2 Authors’ original file for figure 3 Authors’ original file for figure 4

## Abbreviations

BMI: Body mass index
GP: General practitioner
HADS: Hospital Anxiety and Depression Scale
HRQoL: Health-related quality of life
HZ: Herpes zoster
MCS: Mental Component Summary (SF-12)
NPSI: Neuropathic Pain Symptom Inventory
NS: Non-significant
PCS: Physical Component Summary (SF-12)
PHN: Post-herpetic neuralgia
SD: Standard deviation
SF-12: Short-Form health survey
VZV: Varicella-zoster virus
ZBPI: Zoster Brief Pain Inventory.
