# Supplementary figures and images for: ARIZONA study: is the risk of post-herpetic neuralgia and its burden increased in the most elderly patients?
Source: BMC Infect Dis. 2014 Oct 1;14:529. doi: 10.1186/1471-2334-14-529 (PMC4261572; doi:10.1186/1471-2334-14-529)

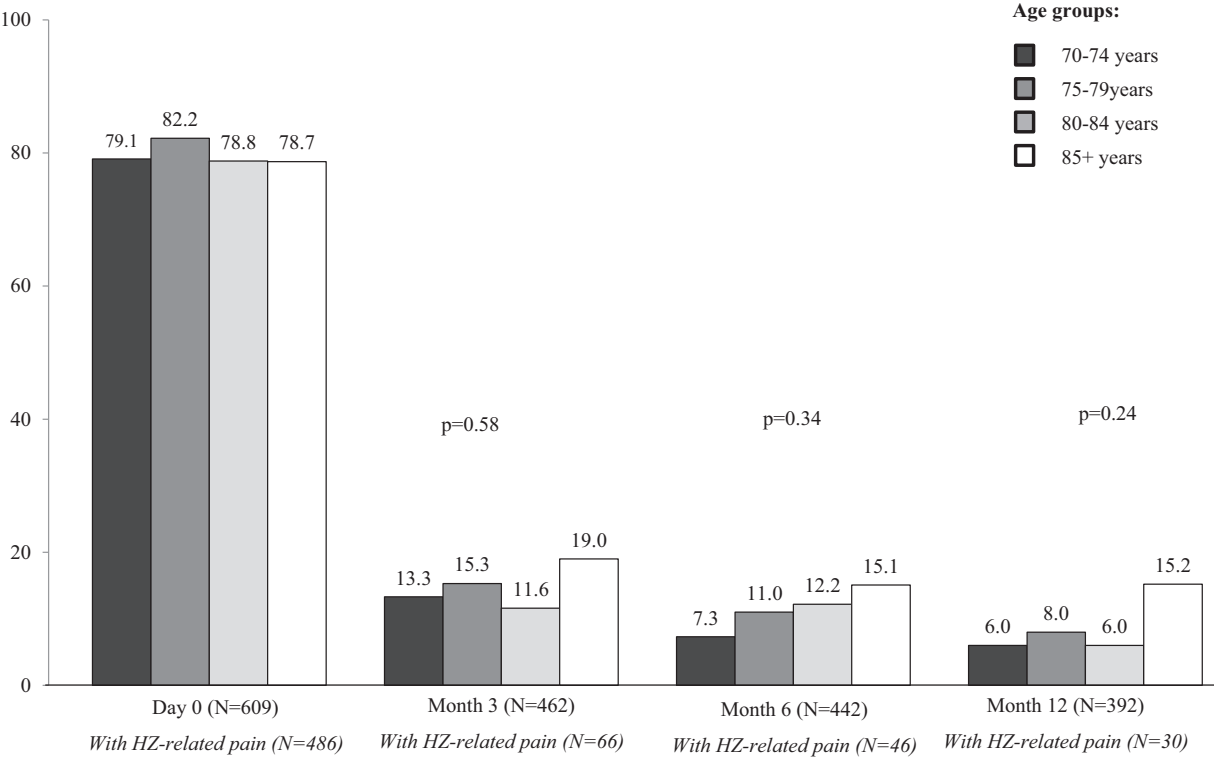

Supplement: Supplementary file 1 — Authors’ original file for figure 1 [file 12879_2014_3849_MOESM1_ESM.pdf]

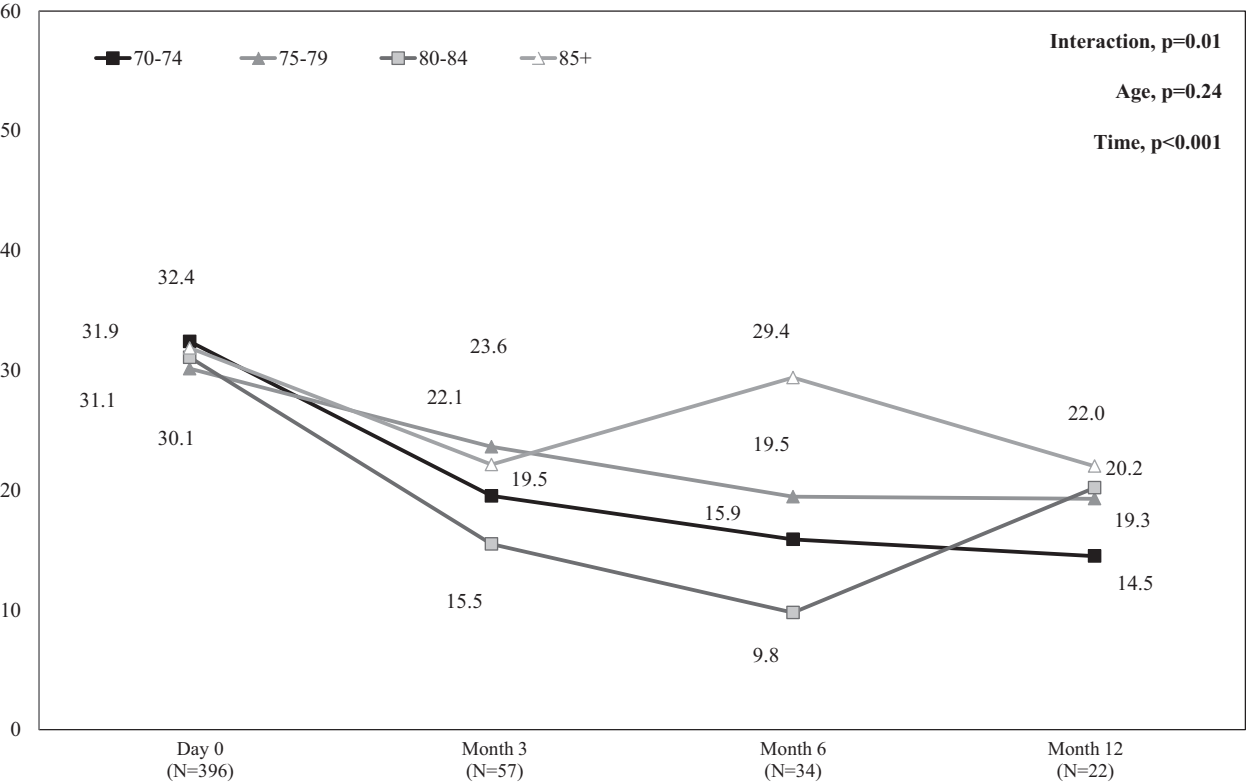

Supplement: Supplementary file 2 — Authors’ original file for figure 2 [file 12879_2014_3849_MOESM2_ESM.pdf]

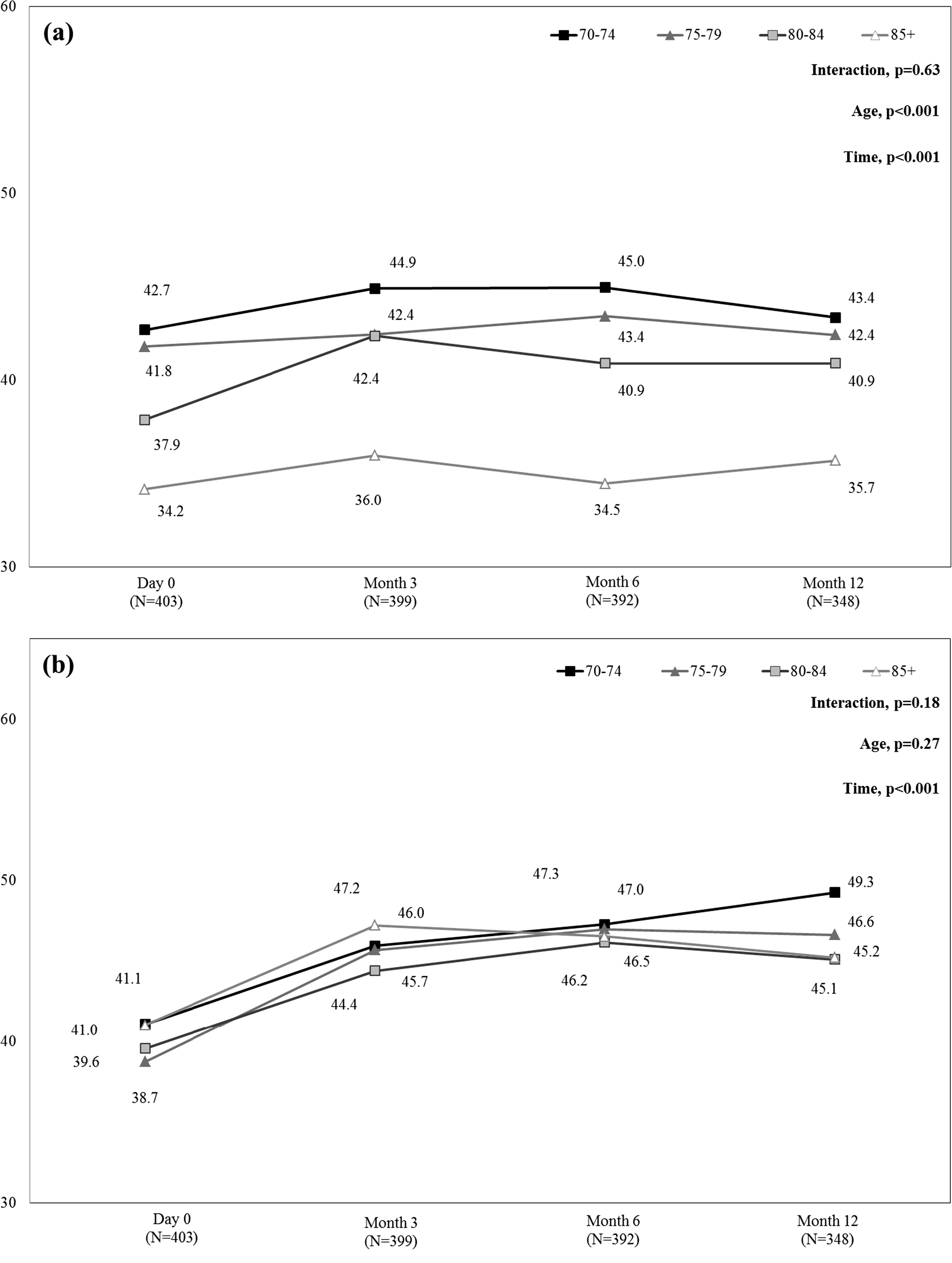

Supplement: Supplementary file 3 — Authors’ original file for figure 3 [file 12879_2014_3849_MOESM3_ESM.tif]

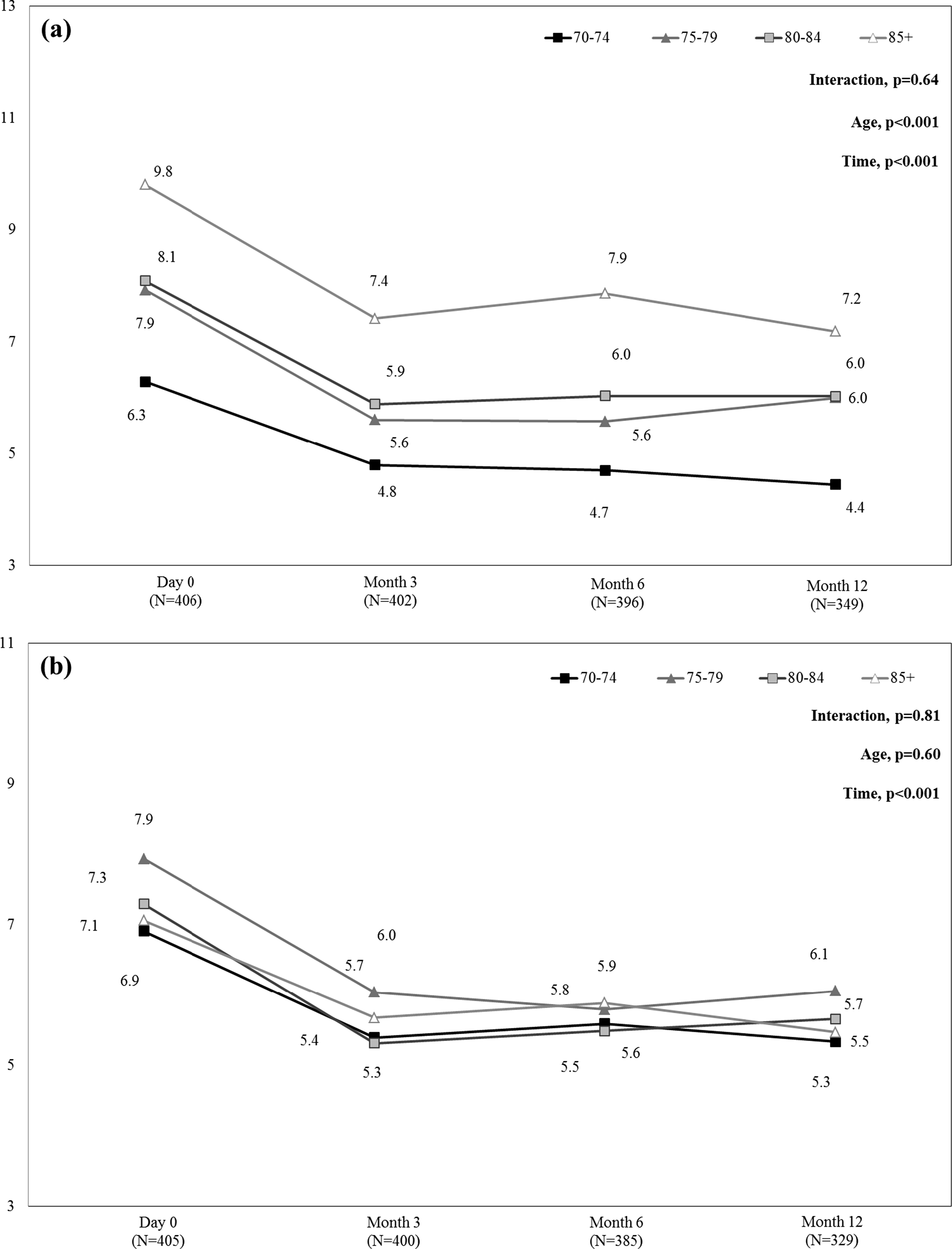

Supplement: Supplementary file 4 — Authors’ original file for figure 4 [file 12879_2014_3849_MOESM4_ESM.tif]
